# Systematic identification and expression profiles of the BAHD superfamily acyltransferases in barley (*Hordeum vulgare*)

**DOI:** 10.1038/s41598-022-08983-7

**Published:** 2022-03-24

**Authors:** Zhen Yuan, Hongliang Yang, Leiwen Pan, Wenhui Zhao, Lunping Liang, Anicet Gatera, Matthew R. Tucker, Dawei Xu

**Affiliations:** 1 School of Agronomy, Anhui Agricultural University, Hefei, 230036 China; 2 School of Agriculture, Food and Wine, Waite Research Institute, University of Adelaide, Adelaide, SA 5064 Australia

**Keywords:** Functional clustering, Plant cell biology, Plant evolution

## Abstract

BAHD superfamily acyltransferases play an important role in catalyzing and regulating secondary metabolism in plants. Despite this, there is relatively little information regarding the BAHD superfamily in barley. In this study, we identified 116 HvBAHD acyltransferases from the barley genome. Based on phylogenetic analysis and classification in model monocotyledonous and dicotyledonous plants, we divided the genes into eight groups, I-a, I-b, II, III-a, III-b, IV, V-a and V-b. The Clade IV genes, including *Agmatine Coumarol Transferase* (*ACT*) that is associated with resistance of plants to Gibberella fungi, were absent in Arabidopsis. Cis-regulatory element analysis of the *HvBAHDs* showed that the genes respond positively to GA3 treatment. In-silico expression and qPCR analysis showed the *HvBAHD* genes are expressed in a range of tissues and developmental stages, and highly enriched in the seedling stage, consistent with diverse roles. Single nucleotide polymorphism (SNP) scanning analysis revealed that the natural variation in the coding regions of the *HvBAHDs* is low and the sequences have been conserved during barley domestication. Our results reveal the complexity of the *HvBAHDs* and will help facilitate their analysis in further studies.

## Introduction

**Figure 1:**
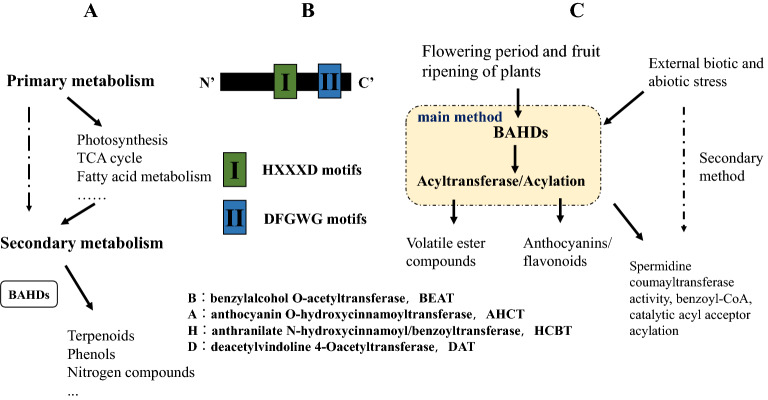
Functional roles of *BAHD* genes in plants. (**a**) *BAHDs* play a prominent role in secondary metabolism. (**b**) Typical protein structure of BAHDs. (**c**) Activation of BAHDs.

Acyltransferases have multiple functions, fulfilling an important role in plant gene expression, metabolism, and signal transduction. Plant secondary metabolism results in the production of many important metabolites, such as phenols (flavonoids), isoprene compounds (such as terpenoids) and nitrogen-containing compounds (such as alkaloids) (Fig. 1a). The BAHD acyltransferases catalyze the acylation of many plant secondary metabolites. They represent a superfamily of enzymes found primarily in plants, algae and bacteria that are responsible for transfer of acylated moieties (RC(O)R’) from an acyl-activated CoA thioester donor to an acceptor molecule, whose products include small volatile esters, modified anthocyanins as well as constitutive defense compounds and phytoalexins^1^. BAHD acyltransferases were named after the first letter of each of the first four enzymes identified biochemically in this superfamily^2^. These four enzymes are BEAT benzylalcohol O-acetyltransferase (BEAT), cloned from *Clarkia breweri* and involved in the production of volatile ester compounds, anthocyanin O-hydroxycinnamoyltransferase (AHCT), cloned from *Gentiana triflora* and involved in the acylation of anthocyanin, anthranilate N-hydroxycinnamoyl/benzoyltransferase (HCBT), cloned from *Dianthus caryophyllus* and involved in the production of anthramide plant antitoxins, and deacetylvindoline 4-Oacetyltransferase (DAT) cloned from *Catharanthus roseus*, involved in the final step of the synthesis of the alkaloid vindoline, deacetylated vindoline 4-O-acetyltransferase. Among these enzymes there are two kinds of acyltransferases, one that mainly acts on the release of plant volatile ester compounds, and another that is closely related to the synthesis of plant anthocyanins/flavonoids. Members of the HCBT and DAT families typically share two important conserved amino acid motifs including HXXXD (Histidine, XXX, Aspartic acid) that is typically found in the central region of the enzyme, and DFGWG (Aspartic acid, Phenylalanine, Glycine, Tryptophan, Glycine), a conserved motif that is typically found near the carboxyl terminus of the protein and is thought to participate in CoA binding (Fig. 1b)^1^.

In recent years, based on the completion of rice and *Arabidopsis* whole genome sequencing, at least 119 rice genes have been identified as part of the *BAHD* superfamily, and 64 (including pseudogenes) have been identified in Arabidopsis^3^. This has expedited research around *BAHD* gene function, catalytic mechanisms, evolutionary relationships, and in particular, their relevance to medicine. Polyphenolic compounds are an important dietary component, and therapeutic benefits of hydroxycinnamate ester conjugates such as Monolignol hydroxycinnamate have been discussed^4^. Similarly, a BAHD enzyme Feruloyl-CoA:monolignol Transferase (AsFMT) from Chinese angelica (*Angelica sinensis*) is involved in the production of coniferyl ferulate, which has antibacterial, antioxidant and anti-Alzheimer’s properties^5^. It has also shown potential reversal effect in antitumor adjuvant therapy^6^. The formation of chicory acid in echinacea also involves BAHD acyltransferase; this metabolite has high medicinal value in antiviral, anti-inflammatory, and glycolipid balance^7^.

The most common way for eukaryotic proteins to be modified after translation is via acylation or deacylation, and this is controlled by the abundance and activity of acyltransferase enzymes. These enzymes can have diverse roles in growth and development. Several characterized BAHD members in *Arabidopsis* use hydroxycinnamoyl-CoAs as substrates, including Hyroxycinnamoyl-Coenzyme A: Shikimate/Quinate Hydroxycinnamoyltransferase (HCT), which is involved in lignin synthesis^8^, the Spermidine Hydroxycinnamoyl Transferase (SHT), which is involved in the synthesis of hydroxycinnamoyl spermidines in the tapetum of *Arabidopsis* anthers^9^, and polyamine acyltransferase, responsible for the accumulation of spermidine conjugates in *Arabidopsis* seed^10^. Acetyl CoA:(Z)-3-hexen-1-ol Acetyltransferase (CHAT) is responsible for producing the green leaf volatile (Z)-3-hexen-1-yl acetate in *Arabidopsis thaliana*^11^, while *Defective in Cuticular Ridges* (*DCR*) is required for incorporation of the most abundant monomer into the polymeric structure of the *Arabidopsis* flower cutin^12^. There have also been reports of suberin and cutin feruloyl transferases and a wax fatty alcohol caffeoyl transferase, which transfer hydroxycinnamoyl-CoAs to ω-hydroxy fatty acid acceptors that accumulate in the extracellular matrix^13–15^. Recently a novel BAHD family acyltransferase *Brassinosteroid Inactivator2* (*BIA2*) was identified that is involved in BR homeostasis and may inactivate bioactive BRs by esterification, particularly in roots and hypocotyls under dark conditions^16^. In rice, BAHD acyltransferase OsAT10 functions as a p-coumaroyl coenzyme A transferase involved in glucuronoarabinoxylan modification^17^, while the *Defective Pollen Wall 2* (*DPW2*) BAHD acyltransferase is important for the formation of rice pollen wall; after knockout of DPW2, the pollen wall is destroyed, leading to pollen abortion (Fig. 1c)^18^. Acyltransferases can also catalyze the esterification of cholesterol (acyl-CoA: cholesterol acyltransferase, ACAT)^19^, catalyzing free cholesterol to form cholesterol ester.

Barley (*Hordeum vulgare*) is the fourth major cereal in terms of production just after maize, rice and wheat, and has high value in terms of animal feed, human food, and brewing industries. Barley also has the advantages of strong resistance to stress, strong adaptability, and wide-ranging cultivation. The International Barley genome Sequencing Consortium (IBSC) recently released the first version of the barley reference genome sequence^20^, and with the sequencing of the barley pan-genome^21^, considerable genetic information has been unearthed. At present, the genome-wide analysis of the barley *BAHD* gene family has not been reported. Such an analysis would provide scope to investigate interspecific similarities and putative unique functions in the Triticeae cereals. Here, we mined genomic sequence resources and constructed a Hidden Markov Model (HMM) of the barley *BAHD* gene family. This was used this to define the *BAHD* supergene family, assess gene expression and predict putative functions of barley *BAHD*-related genes^22,23^.

## Results

### Identification of *HvBAHD* genes in the barley genome

The genome sequence of Barley cultivar Morex was used to identify putative *HvBAHD* genes. To achieve this, local protein BLASTs were carried out using HvBAHD protein sequences from *Oryza sativa* and *Arabidopsis thalian*a. In parallel, an HMM search of the Pfam website was performed using the conserved HvBAHD superfamily feature domain (Pfam:PF02458). A total of 116 genes were identified from *Hordeum vulgare* after deleting redundant sequences and designated as *HvBAHD001* to *HvBAHD116*. Almost all selected genes encode the HXXXD domain, but the DFGWG domain showed variability. The basic physical and chemical properties of the putative HvBAHD proteins such as amino acid length, molecular weight (Da), isoelectric point (pI), hydrophobicity prediction, and GC (%) content are summarized (Supplementary Table 1). The length of 75% of the HvBAHD proteins is around 412–499 amino acids, which is the same as *Arabidopsis* and rice, 63% (73/116) are hydrophilic, 72% exhibit an isoelectric point less than 7, and the molecular weight ranged from 40 to 59 kDa. The Gene ID for each sequence derived from Golden Promise is provided in Supplementary Table 1^24^.

### Phylogenetic analysis of HvBAHD proteins

**Figure 2:**
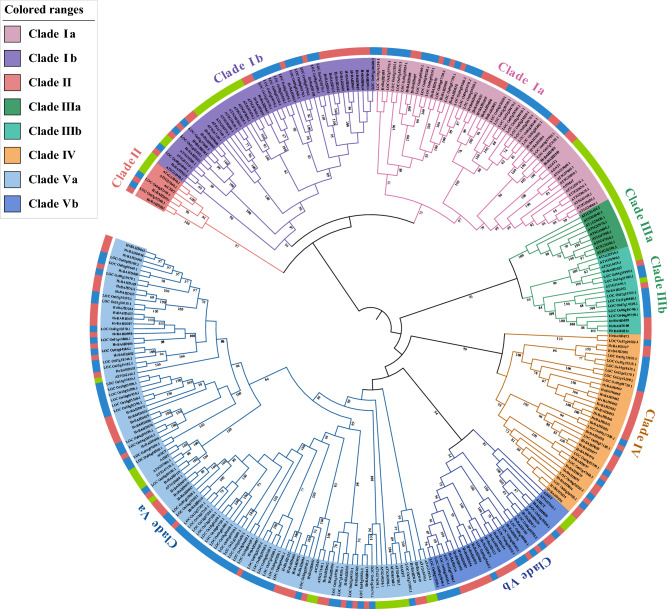
Phylogenetic analysis of BAHD proteins. The BAHD sequences of *Arabidopsis thaliana*, rice and barley were used to construct a phylogenetic tree using the maximum likelihood method. The sequences were divided into five clades. Different clades in the inner ring are distinguished by different colors, and different colors in the outer ring represent different species (green is Arabidopsis, red is barley, blue is rice). This phylogenetic tree was generated by MEGA X (https://www.megasoftware.net/) and iTOL online site (https://itol.embl.de/).

To investigate the cross-species relationship of BAHD family proteins, phylogenetic evolutionary trees of 295 BAHD protein sequences (116 barley, 119 rice and 60 Arabidopsis) are constructed using MEGA X (https://www.megasoftware.net/), and the phylogenetic trees was generated by the maximum likelihood method. The protein sequences of the HvBAHD family proteins were classified according to the classification of rice and Arabidopsis following previous methods^1,3,25^. According to the clustering with known Arabidopsis and rice orthologs, barley BAHDs proteins were divided into 5 clades (Fig. 2, Supplementary Fig. S1).

Clade I consists of two subclades: Clade Ia (*15 HvBAHDs*) and Clade Ib (18 *HvBAHDs*) (Fig. 2) while Clade II (2 *HvBAHDs*) contains the fewest members (Fig. 2). Clade III can be divided into two subclades: Clade IIIa (0 *HvBAHDs*) and Clade IIIb (5 *HvBAHDs*) (Fig. 2). There are two genes showing homology to Eceriferum2 (CER2) in barley, *HvBAHD031* and *HvBAHD083* (Fig. 2), which appear highly conserved compared to rice and Arabidopsis. Clade IV (22 *HvBAHDs*) appears to be specific to the monocot species analyzed here (Fig. 2). One of the BAHD proteins in this clade, the agmatine coumarol transferase (ACT), is reported to catalyze the synthesis of hydroxycinnamate agmatine from barley agmatine. Hydroxycinnamoyl-CoA thioester, its precursor, can act as an antifungal compound hordatine^26,27^. Hydroxycarnitine agmatine derivatives have also been found at low levels in wheat^28^. Clade IIIa may be specific to dicots, as rice and barley sequences are absent. AT4G15400 in this clade is involved in the synthesis and modification of the triterpene analogue brassinosteroid (BR), and thus participates in the regulation of BR homeostasis and plant light signals^29^. Although most members in Clade III have different acylated alcohol acceptors, their donors are predominantly acetyl-CoA. Some acetyl-CoA BAHDs are involved in the synthesis of volatile esters in fruit (e.g., *Rosa chinensis* RhAAT1), and others are involved in the modification of alkaloids. Clade V incorporates the largest number of *HvBAHDs* and can be divided into two subclades, Clade Va (39 *HvBAHDs*) and Clade Vb (15 *HvBAHDs*). In Clade Va, *HvBAHD029* is the likely barley orthologue of *AT5G41040* (ASFT), *AT5G63560* (FACT) and *AT3G48720* (DCF), which are involved in suberin and cutin biosynthesis^13,30–32^. Also in this clade, *HvBAHD115* is the orthologous gene of *AT2G23510* (spermidine diprosyl acyltransferase; SDT), which is required for the production of disinapoyl spermidine and its glucoside in seeds, and *AT2G25150* (spermidine dicoumarolyl acyltransferase SCT), which is mainly expressed in roots and has spermidine coumaroyl CoA acyltransferase activity^33^. The characteristic protein of acyl CoA in Clade Va is involved in the formation of volatile esters and is related to hydroxycinnamoyl transferase (HCT) which is responsible for the synthesis of chlorogenic acid and monoxylitol. AtHCT (*AT5G48930*) is in Clade V, which includes *HvBAHD011*, *HvBAHD010* and *HvBAHD082*^17^. Bartley et al., and others indicate that HCT is involved in the synthesis of lignin precursors in most vascular plants, since several characterized members (e.g., hydroxycinnamoyl-coenzyme A shikimate/quinate HCT) use hydroxycinnamoyl-CoAs as substrates. HQT is closely related to HCT, both which have HHLVD and DFGWG motifs.

In general, close barley homologues of functional BAHD genes are present in every sub-clade, except for Clade IIIa. This indicates that their function as acyltransferases in secondary metabolism may have been retained through evolution.

### Chromosomal location of *BAHD* genes

**Figure 3:**
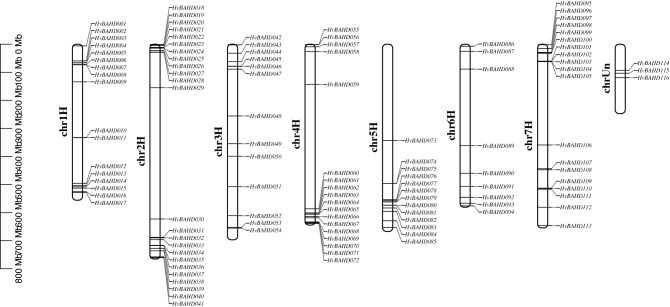
Distribution of 113 putative *HvBAHD* genes (3 genes on unknown chromosome; chrUn) across the seven chromosome (chr) groups of barley. All barley chromosomes are drawn to scale based on predicted lengths. This picture was constructed by TBtools v1.082 (https://doi.org/10.1016/j.molp.2020.06.009).

All 116 *HvBAHDs* genes were mapped onto barley chromosomes, designated as *HvBAHD001* to *HvBAHD113* according to the physical location, except for *HvBAHD114* to *HvBAHD116* whose locations remained unknown (Fig. 3). Further, chromosome position analysis showed that these genes were unevenly distributed on most chromosomes (chr) of barley. Chromosomes 2, 7, 4 and 1 contained the majority of *BAHD* genes (Fig. 3), which were distributed mainly at the ends of the chromosomes.

### Analysis of conserved motif and cis-acting elements in the promoters of *HvBAHD* genes

**Figure.4:**
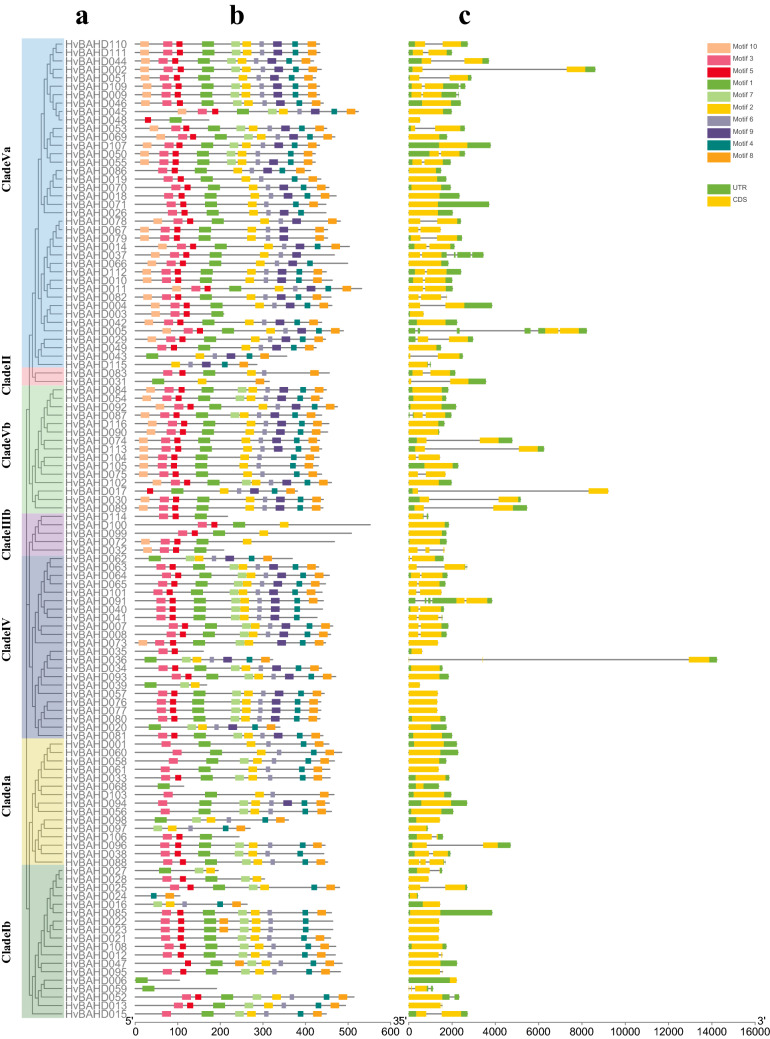
Phylogenetic relationships, gene structure and architecture of conserved protein motifs in *BAHD* genes from barley. (**a**) A maximum likelihood tree was constructed using the predicted amino acid sequences of HVBAHD proteins. This phylogenetic tree was generated by MEGA X (https://www.megasoftware.net/) and iTOL online site (https://itol.embl.de/). (**b**) The motif composition of barley BAHD proteins. The motifs are displayed in different colored boxes, motifs in the HvBAHD proteins were elucidated by MEME 5.0.1 (https://meme-suite.org/meme/tools/meme). Motif1 is HXXXD domain and Motif4 is DFGWG domain. The sequence information for each motif is provided in Supplementary Table S2. (**c**) Exon–intron structure of barley *BAHD* genes. The green and yellow squares represent the untranslated region (UTR) and coding sequences (CDS) respectively. The black lines represent the intronic. Genomic structure was constructed by TBtools v1.082 (https://doi.org/10.1016/j.molp.2020.06.009).

Protein motif distributions may contribute to functional diversity among the members of a gene family. Therefore, we assayed the motif distribution in predicted full length protein sequences of HvBAHD proteins using MEME (Fig. 4; Supplementary Table S2). A total of 10 motifs were predicted. Most HvBAHDs contained both motif1 and motif4. Based on gene structural analysis we determined that motif1 and motif4, with sequences FTCGGFVIGLRTNHAVADGTGAAQFLNAV and FDVYGNDFGWGRPV, correspond to domains HXXXD and DFGWG, respectively (Supplementary Table S2). The type and distribution of conserved motifs in the same subfamily of HvBAHDs are similar, which is consistent with the clade classification results.

To assess conservation of putative cis-regulatory elements in the *HvBAHD* genes we assessed all 116 genes to identify 2000 bp of upstream sequence. However, the barley intergenic sequence is currently interspersed with small regions of missing data. To ensure the reliability of prediction, we ignored *HvBAHDs* with missing sequence data and focused on 79 *HvBAHDs* containing complete upstream 2000-bp sequences. Sequences from each clade of *HvBAHDs* were represented in the 79 upstream regions, so the results were general to some extent, but not completely representative of the *HvBAHD* family. The results show that some cis-regulatory elements such as photo-responsive elements, plant hormone responsive elements, plant defense, and compressive stress were all related to putative *HvBAHD* family function. Details are shown in Supplementary Table S3. The two fundamental gene elements of the CAAT-BOX (Common Cis-Acting Element in Promotor and Enhancer Regions) and TATA-BOX (Core Promotor Element Around-30 of Prescription Start) were removed, and the remaining elements were plotted (Supplementary Fig. S2). Certain elements were clearly prevalent in a majority of *HvBAHDs*, include light-responsive (G-box), biotic and abiotic stress-responsive elements (As-1, ARE, STRE, MYB and MYC) which were identified at a high frequency. These elements are also found in *Arabidopsis thaliana* BAHD sequences but without special annotation information. It is possible they reflect important conserved elements of the BAHD family. The cis-element analysis identified several gibberellin-related reaction elements in the *HvBAHD* genes that may contribute to activation of *HvBAHD* genes. Moreover, previous studies have shown that the ACT-related genes in barley antagonize gibberellin function.

### Evolutionary analysis of *HvBAHD* genes

**Figure 5:**
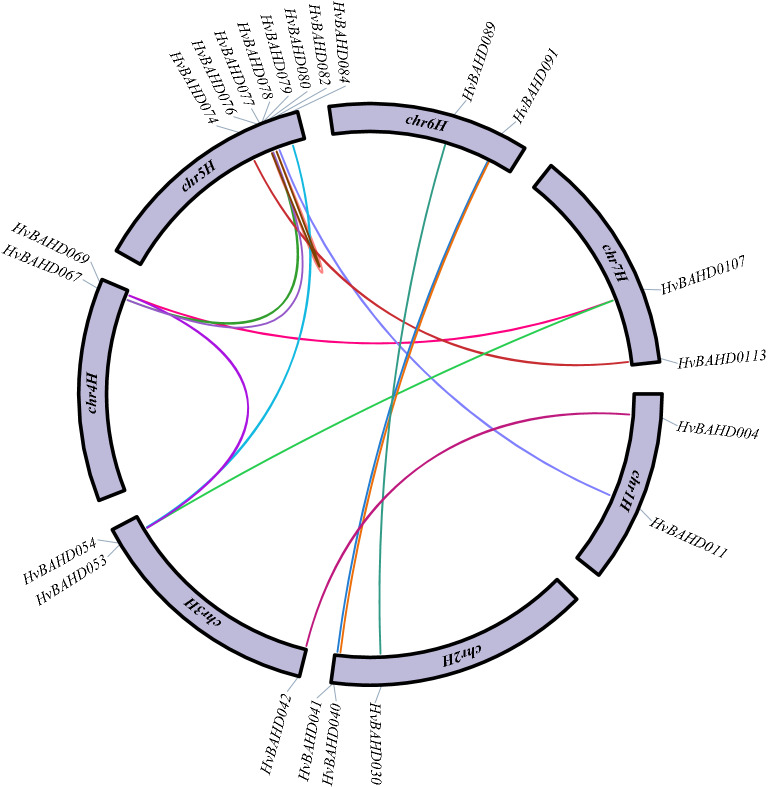
The homologous gene pairs and chromosomal locations of the barley *BAHD* members. Lines of different colors represents a certain replication relationship between the two genes. The outer circle is the gene name of the associated gene and the distribution position on the chromosome. This picture was constructed by TBtools v1.082 (https://doi.org/10.1016/j.molp.2020.06.009).

Phylogeny can often reveal information regarding gene duplication events. The Ka/Ks value is an important parameter that is used to evaluate the evolution of coding sequences and determine the type of selection pressure after duplication. In our study, we searched for evidence of gene duplication through local blast comparisons of barley *BAHD* genes and screening of data to identify sequences with > 75% identity^34^. According to the phylogenetic analysis, there are 14 pairs of homologous genes in the *HvBAHDs* (Fig. 5); the putative tandem repeat genes were analyzed by KaKs, and the Ks, Ka, and the Ka/Ks ratio for the 14 gene pairs are shown in Supplementary Table S4.

Non-synonymous changes are typically subject to natural selection, while synonymous changes are not. In evolutionary analysis, it is of great significance to understand the rate of occurrence of synonymous and non-synonymous changes. If Ka/Ks > 1, it is considered that there is a positive selection effect, if Ka/Ks = 1, it is considered that there is a neutral choice, if Ka/Ks < 1, it is considered to have purification selection effect^35^. Among the 14 selected paralogue gene pairs, the ratio of Ka/Ks of 10 genes pairs was less than 1, which is consistent with synonymous substitution rather than nonsynonymous substitution to prevent the change of amino acid residues after duplication.

### Gene expression analysis of *HvBAHDs* by qRT-PCR and in response to GA3 treatment

**Figure 6:**
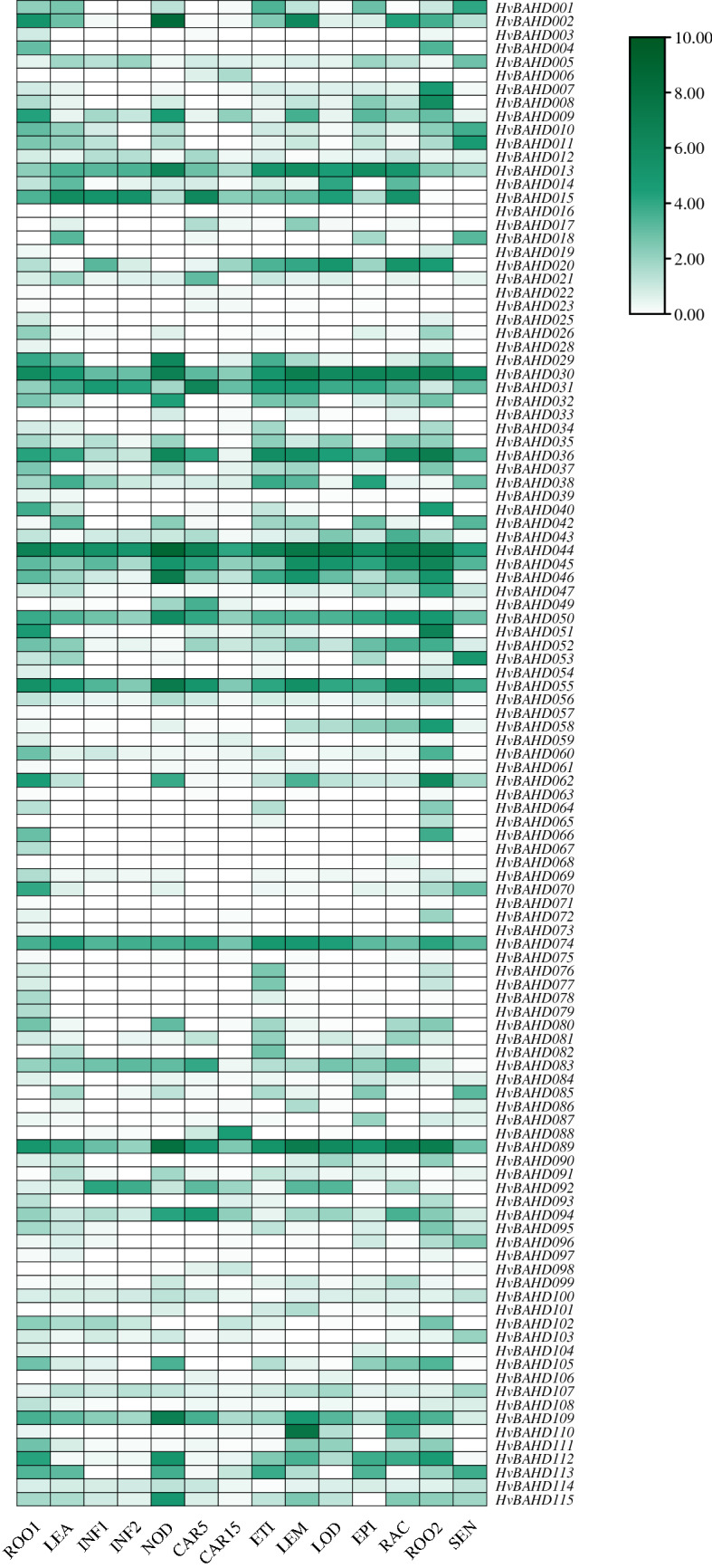
Expression profiles of a subset of *BAHD* genes in different barley tissues and organs. The transcript levels are indicated by a graded color scale from white to green. Expression data were obtained from IPK (https://www.ipk-gatersleben.de/). Normalized expression levels were transformed by logarithmic function and hierarchical clustering of the subset of *HvBAHD* genes are shown. The heatmap is drawn by the TBtools v1.082 (https://doi.org/10.1016/j.molp.2020.06.009). ROO1: Roots from seedlings (10 cm shoot stage); LEA: Shoots from seedlings (10 cm shoot stage); INF1: Young developing inflorescences (5 mm); INF2: Developing inflorescences (1–1.5 cm); NOD: Developing tillers, 3rd internode (42 DAP); CAR5: Developing grain (5 DAP); CAR15: Developing grain (15 DAP); ETI: Etiolated seedling, dark cond. (10 DAP); LEM: Inflorescences, lemma (42 DAP); LOD: Inflorescences, lodicule (42 DAP); EPI: Epidermal strips (28 DAP); RAC: Inflorescences, rachis (35 DAP); ROO2: Roots (28 DAP); SEN: Senescing leaves (56 DAP).

To analyze the expression patterns of the barley *BAHD* gene family, expression data from different stages, organs and tissues were extracted from the IPK website and examined using a hierarchical clustering model. The heatmap shows that 111 HvBAHDs were expressed in 15 different developmental stages based on web RNA-seq data (Fig. 6). The *HvBAHDs* were expressed to different degrees at all stages of barley growth. Generally, it can be seen that the expression level of *HvBAHDs* in the older roots is higher than young roots, and the expression level of several genes which belong to Clade V (like *HvBAHD030*, *HvBAHD044*, *HvBAHD055*) was high at all stages (Fig. 6).

**Figure 7:**
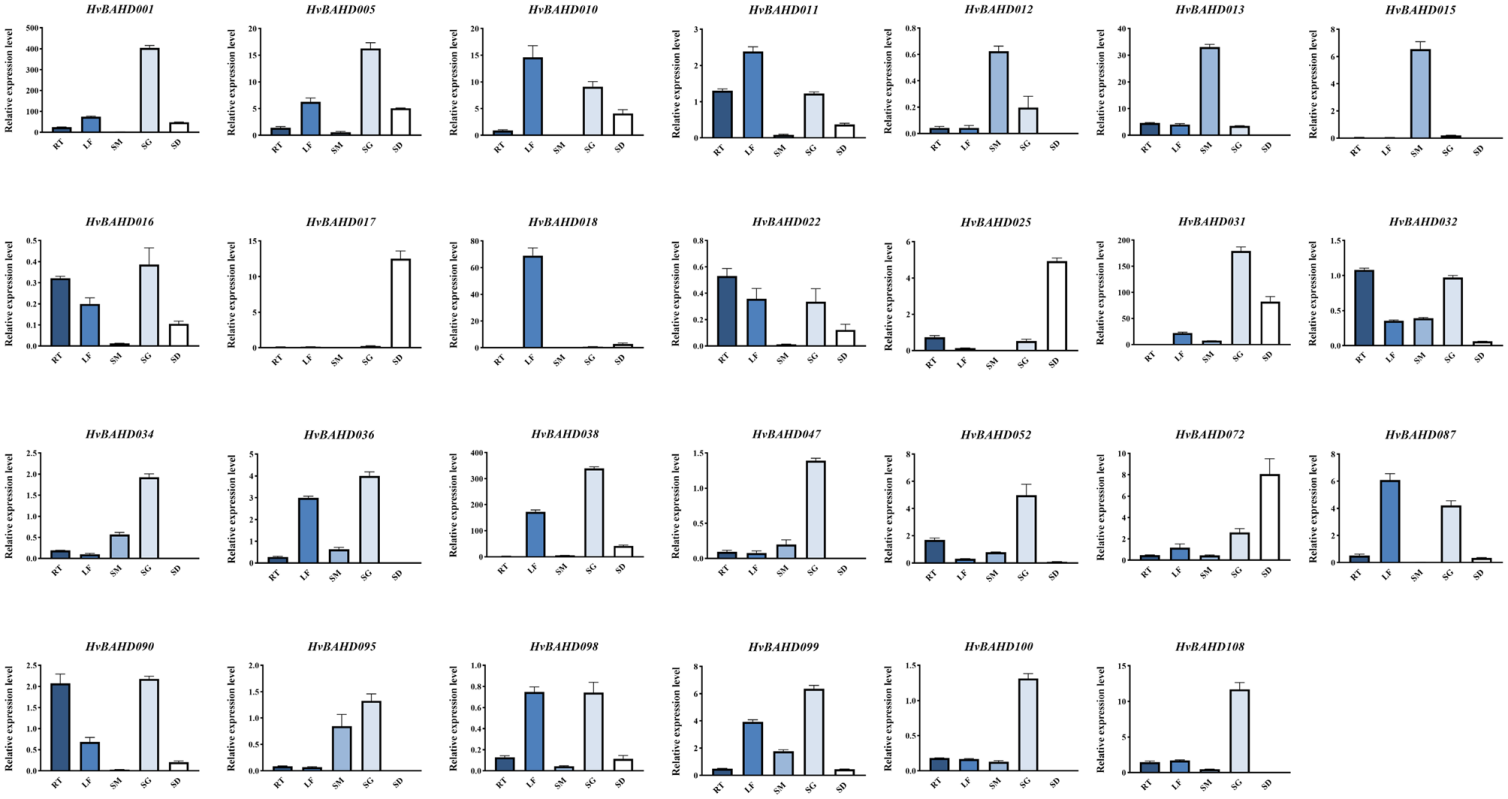
The relative expression levels of the *HvBAHD* genes in five different barley tissues as determined by qRT-PCR. RT: root; SM: stem; LF: leaf; SG: 2-week-old seedling; SD: ripe seed. This picture was generated by GraphPad 8 (https://www.graphpad-prism.cn/) .

To better understand the potential roles of *HvBAHDs* in different tissues, we performed qRT-PCR on 27 genes from 5 clades. The results showed that *HvBAHD012*, *HvBAHD013*, *HvBAHD015* and *HvBAHD095* from Clade I were highly expressed in stems (Fig. 7). *HvBAHD034* and *HvBAHD036* from Clade IV showed high expression at the seedling stage, (Fig. 7). *HvBAHD032*, *HvBAHD099* and *HvBAHD100* distributed in Clade IIIb, and many genes in this clade are involved in the formation of volatile substances and alkaloids; qRT-PCR showed that these genes were highly expressed not only in young leaves but also in roots (Fig. 7). *HvBAHD052* shares homology with genes involved in the synthesis of hydroxycinnamyl transferase (HCT) and is expressed in young leaves (Fig. 7). The qRT-PCR analysis showed that many of the *BAHD* genes are expressed in developing seedlings. However, there are clear differences in gene expression from different clades, which may relate to different functional roles in specific tissues within the BAHD family.

**Figure 8:**
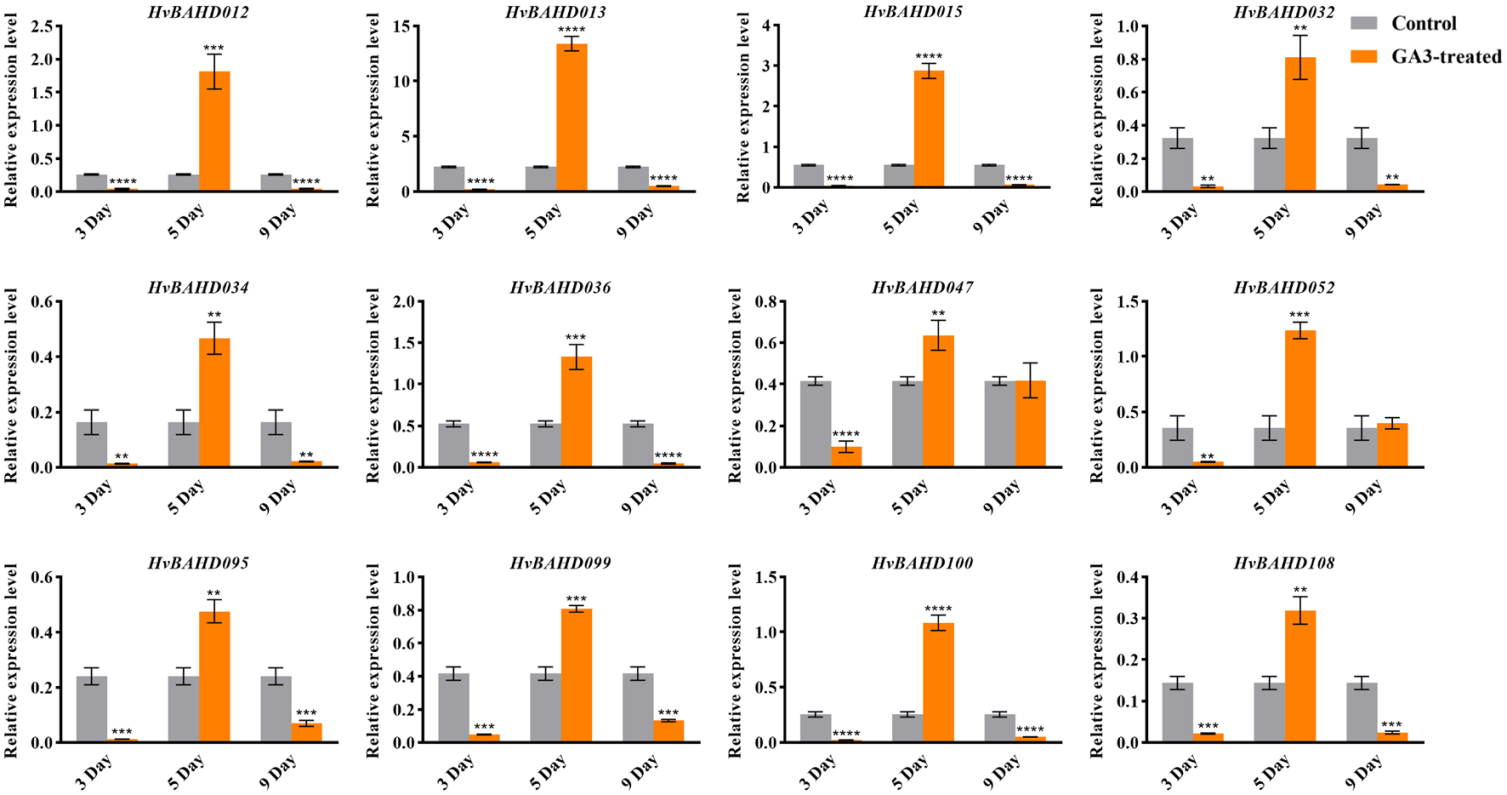
Relative expression level of *HvBAHD* genes on the 3rd, 5th and 9th day after GA3 treatment in barley leaves. Gray shows the control group and orange indicates the GA3 treatment group. (P < 0.0001, ****, P < 0.001, ***, P < 0.01, **, P < 0.1, *). This picture was generated by GraphPad 8 (https://www.graphpad-prism.cn/) .

The cis-element analysis identified several GA-responsive elements in the promoters of selected *BAHD* genes, which may relate to the role in response the GA pathway. For example, GARE-motif and p-box (Gibberellin-responsive element) are related to gibberellin response in barley and were particularly prevalent in Clade IV genes. ACT in Clade IV was related to Gibberella zeae, while there was a subtle relationship between Gibberella zeae and gibberellin. To test this further, transcriptional responses of selected *HvBAHD* genes were examined in plants containing 5 leaves after treatment with 50 mg/L GA3 for 3 days, 5 days, and 9 days. The results of qRT-PCR are shown in Fig. 8. After treatment of leaves with GA3, we found that the expression of *HvBAHD* genes did not respond significantly on the 3rd day after treatment, but the gene expression was increased significantly after the 5th day of treatment (Fig. 8).

**Figure 9:**
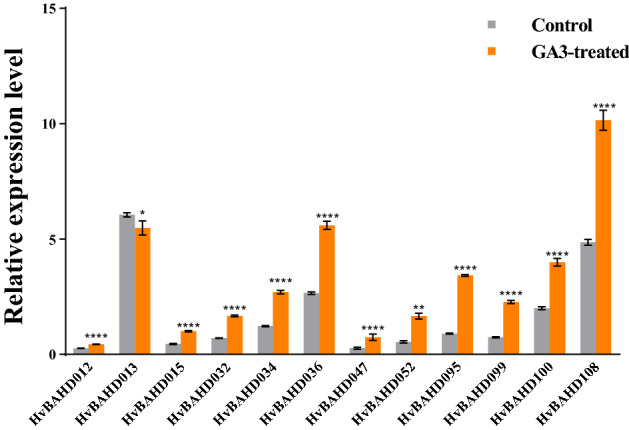
Relative expression of *HvBAHD* genes after 5 days of GA3 treatment in barley seedlings. Gray was used as the control group and orange was used as the GA3 treatment group. (P < 0.0001, ****, P < 0.001, ***, P < 0.01, **, P < 0.1, *). This picture was constructed by GraphPad 8 (https://www.graphpad-prism.cn/) .

The high level of expression level of *HvBAHD* genes at seedling stage was also of interest, so we conducted another GA3 spray treatment on the seedlings. Leaves were collected from seedlings sprayed with GA3 or water after 5d. qRT-PCR showed that most *HvBAHD* genes assayed showed a significant increase in expression, indicating that GA3 had already affected the plants and *HvBAHD* genes were responding (Fig. 9).

### Subcellular localization of selected HvBAHDs in barley

**Figure 10:**
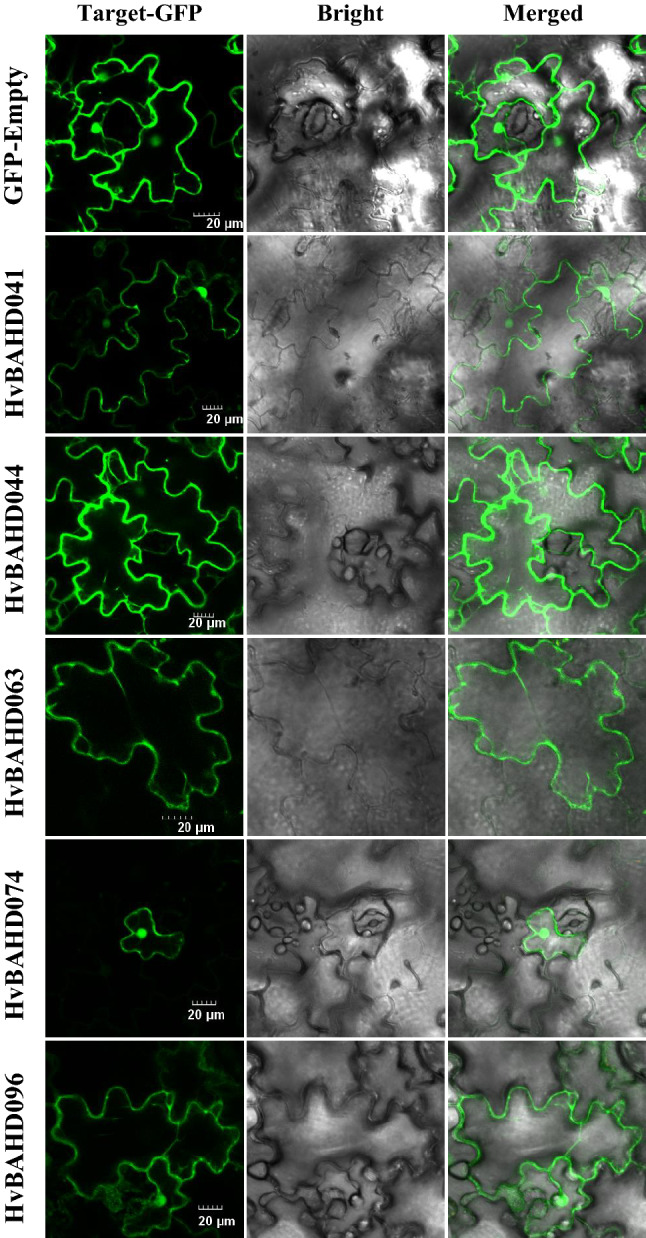
Subcellular localization of HvBAHD proteins in barley. Selected HvBAHD-GFP fusion protein as well as GFP-Empty as the control were independently transiently expressed in tobacco leaves and imaged under a confocal microscope. Bars, 20 μm.

The subcellular localization of selected HvBAHD proteins was analyzed to assess possible differences between BAHD proteins in barley. HvBAHD041, HvBAHD044, HvBAHD063 and HvBAHD096 are in the cytoplasm of tobacco (Fig. 10). HvBAHD041 and HvBAHD063 belong to Clade IV, which appears to be a monocot-specific clade potentially linked to anti-fungal function. The biosynthetic pathway related to the Clade IV *ACT* gene in barley is therefore likely to occur within the cells. *HvBAHD096* is a representative gene from Clade Ia, which also contains *Arabidopsis At5MaT* that functions as an anthocyanin 5-glucoside malonyl CoA acyl transferase both in vitro and in vivo^36^. The localization results indicate that the transferases from At5MaT classes are most likely synthesized in the cytoplasm in barley. By contrast, HvBAHD074 is predicted to be predominantly involved in the synthesis of acetyl-CoA (Clade V branch), and localization studies indicate it is in the nucleus.

### SNP data analysis of barley

To investigate the natural variation within the *HvBAHD* family during domestication, we utilised the IPK Gatersleben barley pan-genome database to search for SNPs, and collected information for 93 BAHD genes. Of these, only 52% (48/93) contained any SNPs in the varieties sampled in the SNP-browser, and 47% (44/93) exhibited amino acid changes (Supplementary Fig. S3). However, we also found that the proportion of genes which have SNPs in Clade IV is very high, reaching 71% (12/17), and the two barley genes retrieved in the Clade II sub-clade showed SNP mutation. This indicates that the members of these subunits may have undergone important mutations, and are "young genes" in evolution with relatively high change frequency. Only 32% (30/93) of the *HvBAHD* genes with MAF > 0.05 in SNPs were due to its own functional limitation, which also indicated that most genes were conservative and not affected by SNP site mutations in evolution.

## Discussion

Through the retrieval, analysis and organization of the *BAHD* gene family in barley, a total of 116 *HvBAHDs* were identified, all of which have HXXXD characteristic domains. The total number of genes is similar to that of rice and poplar, which indicates that the *BAHD* family plays an important role in plant evolution. A phylogenetic tree was constructed using the maximum likelihood method, which included 116 barley, 60 *Arabidopsis thaliana*, and 119 rice BAHD protein sequences. During phylogenetic analysis, the 116 *HvBAHDs* were divided into five clades, which were similar to those in rice and Arabidopsis except that Clade IV was specific to Gramineae (Fig. 2). The sequence and molecular weight of the HvBAHDs varied considerably, but the characteristic domains and motif composition were conserved (Supplementary Table 1). The exon/intron composition analysis showed that the number of introns in *HvBAHDs* varied from 0 to 5, but most of the *HvBAHDs* only possess one or no introns. The number of introns in different groups was remarkably conserved, except for *HvBAHD005* and *HvBAHD037* (Fig. 4), similar to other plant species^1,25,37^. We found that most of the *HvBAHDs* are distributed towards the gene-rich ends of the chromosomes (Fig. 3), similar to reports for other barley gene families^38,39^. Gene duplication is one of the major mechanisms contributing to genome complexity, as well as to the rapid expansion and evolution of gene families^40,41^. Gene chromosomal distribution demonstrates that at least 14 pairs of *HvBAHDs* have undergone gene duplication (Fig. 5; Supplementary Table S4), and most of these gene duplication events correspond to segment duplication. Only two pairs of *HvBAHDs* (*HvBAHD76* and *HvBAHD80*) and (*HvBAHD77* and *HvBAHD80*) are the products of tandem replication (Fig. 5). The segment duplication events of *HvBAHDs* in barley is similar to the previous studies on *BAHDs* of other species^37^.

Most members of the BAHD family are involved in different biochemical reactions, although there are subtle differences from lower plants to higher plants such as BAHD acyltransferase function in various ester synthetic pathways. The *AT4G24510.1* (CER2) located in Clade II contributes to wax formation in the epidermal stratum corneum and physically resists the invasion of external pathogens and controls water loss^42,43^. CER2 have been characterized in *Arabidopsis*, maize, and rice, having similar effects on the extension of waxy VLCFA precursors. CER2 homologues have not been found in *bryophytes*, which suggests CER2 may be a vascular plant innovation^44^. Including these sequences as reference members in our phylogenic analysis provided hints that the HvBAHDs include a large number of potentially novel enzymes, especially for subclades that don’t contain any enzymes of known function (Fig. 2). The phylogenetic tree shows clade IV is a specific clade of Gramineae; there are many related response elements in this branch potentially to improve the function of plants against *Gibberella zeae.* In evolution, a special branch of Gramineae has slowly evolved, and there may be more interesting relationships (Fig. 2). The Agmatine coumaroyltransferase (ACT) in this clade was the first amine N-hydroxycinnamoyltransferase characterized in plants and responsible for catalyze the biosynthesis of antifungal hydroxycinnamoylagmatin derivatives in barley and represents a new class of N-hydroxycinnamoyltransferases^45^. The significant number of HvBAHD proteins in Clade IV suggests a possible role in preventing the invasion of fungi, which may be related to the evolution of barley. Further experiments are needed for the research in Clade IV, potentially in relation to the regulation of gibberellin which has a certain relationship with *Gibberella zeae.*

The cis-regulatory element analysis can provide an important basis for further functional analysis of *HvBAHDs*. In this study, we found that biotic and abiotic stress-responsive elements accounted for the greatest number of cis-regulatory elements predicted in the promoter regions of *HvBAHDs*. This finding indicates the *HvBAHD* genes are likely to participate in responses against various adverse environmental stresses. In addition, GARE-motif and p-box (Gibberellin-responsive element) related to gibberellin response were particularly prevalent in this family. These results are consistent with our GA experiment, which showed that HvBAHDs respond positively to GA3 within a few days of treatment. We also identified several TGACG and CGTCA-motifs, which mainly function as response elements of methyl jasmonate. This is consistent with BAHD function in secondary metabolism and the ability of jasmonate compounds to increase the activity levels of defense proteins such as peroxidase, chitosanase, and lipoxygenase^46,47^. This can result in the accumulation of alkaloids and phenolic acid secondary substances, which increase and change the release of volatile signaling compounds and even form defense structures, such as trichomes and resin conduits. The qRT-PCR results showed that there were expression differences in genes of different branches of *BAHD* family; further experiments are needed to ascertain which members may are associated to JA responses and whether this directly coincides with the presence of cis-elements.

The large number of SNP sites distributed in clade II and clade IV indicated that these two clades may have evolved more specialized functions compared to other branches during plant evolution (Supplementary Fig. S3). Some recent studies have found that the introduction of cell wall BAHD acyltransferase-p-CA monoenol transferase (PMTs) into Arabidopsis and poplar^48^, can promote the quasi-coumarinization of lignin and improve saccharification. This illustrates the importance of BAHD acyltransferases in modifying the cell walls of grasses and dicots, and also illustrates their potential to improve saccharification in feed and bioenergy engineering^49^.

Biochemical functions of specific *BAHD* gene family have now been elucidated, and it is evident that they play an important role in research of various fields. However, the function of many *BAHD* genes remains unclear. This study provides a baseline for further research into the *BAHD* gene family barley, a key representative of the Triticeae cereals, and one for which functional genomics and genetics resources are becoming readily available.

## Conclusion

In this study, the barley BAHD gene family was examined to identify 116 *HvBAHD* genes within the reference genome of cv. Morex*.* The *HvBAHD* genes are present in similar numbers and categories compared to rice and *Arabidopsis,* based on their phylogenetic analysis. The gene structure, MEME, Ka/Ks, and SNP scanning analyses disclose that most of the *HvBAHDs* were highly conserved. However, the Clade IV genes appear to be specific to the monocots, and include the ACT gene that is associated with resistance of plants to Gibberella fungi. Moreover, we investigated promoter sequences of the *HvBAHD* sequences and found the genes respond positively to GA3 treatment. Gene expression analysis identified most genes were expressed in seedlings. To date, no members of BAHD gene family in barley have been characterized in detail. Hence, the results lay a foundation for further research towards addressing the functional characterization, transcriptional regulation, and role of barley BAHD family members.

## Materials and methods

### Source of plants and data

The 2-row spring barley Golden Promise seed was sourced from Shanghai Jiao Tong University. Rice OsBAHD and Arabidopsis AtBAHD protein sequences were downloaded from the Rice Genome Annotation Project (RGAP) (http://rice.plantbiology.msu.edu/)^50^ and The Arabidopsis Information Resource (TAIR) (https://www.arabidopsis.org/), respectively. We obtained the barley nucleotide and protein sequences from the plant genomics database Phytozome (https://phytozome-next.jgi.doe.gov/)^51^. HMMER (https://www.ebi.ac.uk/Tools/hmmer/) was used to retrieve candidate barley protein sequences (E-value < 10^−10^) containing *HvBAHD* conserved domains.

### Sequence identification and collection

TBtools was used to extract the required Rice and Arabidopsis BAHDs protein sequences^52^ and MEGA-X (https://www.megasoftware.net/) was used for alignments^53^. Bio-linux software was used to build HMM with the results^54,55^. The sequences with E-values less than e^−10^ were selected as candidate genes and finally 116 *HvBAHDs* were retained. In order to ensure the reliability of the data, we construct the HMM with the BAHD feature domain (PF02458) provided by Pfam (http://pfam.xfam.org/)^56,57^.

### Phylogenic analysis

The HvBAHDs protein sequences were aligned using ClusterW with default parameters^58^. The phylogenetic tree was constructed with the BAHD proteins from barley, rice and *Arabidopsis* by MEGA-X using the optimal replacement model ML (WAG + G + F) method with 1000 bootstrap replicates^59^. 119 BAHD protein sequences from *Oryza sativa*, 60 BAHD protein sequences from *Arabidopsis thaliana*, and 116 BAHD protein sequences were selected from *Hordeum vulgare*. The constructed phylogenetic tree was rendered using iTOL (https://itol.embl.de/)^60^.

### Gene structure and motif identification

To identify the exon/intron structure of the *HvBAHD* analysis, barley gene CDS and protein sequences were downloaded from the Barley Genome Database Annotation on Phytozome (https://phytozome.jgi.doe.gov/pz/portal.html). The MEME tool was used to search for conserved motifs within HvBAHD proteins (https://meme-suite.org/meme/tools/meme)^61^. The maximum value of the motif was set to 50, and the motif length was set between 6 and 200, the number of motifs is 10, and other parameters by using default settings. Tbtools^52^ was used to analyze the gene structure and conserved motifs. The ExPasy (Compute pI/Mw tool) bioinformatics online tool (https://web.expasy.org/translate/)^62^ was used to predict the isoelectric point (pI) and relative molecular mass (kDa) of each HvBAHD. The 2 Kb upstream region of each *HvBAHD* sequence was chosen for cis-element analysis using the PlantCARE tool^63^ (http://bioinformatics.psb.ugent.be/webtools/plantcare/html/).

### Chromosome position information and collinearity analysis

The predicted location of each gene was collected from the BARLEY IPK website (https://apex.ipk-gatersleben.de/apex/f?p=284:10), and then the chromosome location information was drawn using TBtools. The Ka/Ks were predicted using the KaKs_Calculator2.0^64^ program of Bio-linux^65^.

### Expression analysis of barley *HvBAHD* genes

To explore the specific expression patterns of *HvBAHDs* in different barley tissues and developmental stages, RNA-seq data of different developmental stages were downloaded from IPK (https://galaxy-web.ipk-gatersleben.de/). The gene expression values are represented by fragments per kilobase of exon per million fragments mapped (FPKM). The heatmap is drawn by the online TBtools.

### Plant growth, tissue processing and GA (gibberellin) treatment

The spring barley Golden Promise was cultivated at Anhui Agricultural University (31.85°N, 117.26°E) under normal outdoor temperature (15–25 °C). Seed germination was carried out in early March and seedlings were subsequently transferred to pots. Tissue samples collected from the mature root, stem, leaf, seedling, and spike of barley were wrapped in aluminum foil and dipped into liquid nitrogen. All harvested samples were stored at − 80 °C for future experiments including RNA isolation. For GA3 treatment, 50 ml/L GA3 was applied onto the leaves of five-leaf stage plants. Water spraying was used as the control. In short, two pots of barley plants with representative growth habit were selected. One pot was sprayed with exogenous GA3 (50 mg/L) on the leaves, and the control group was sprayed with water to the same degree. Plants were subsequently sprayed once every other day. Plants were watered and fertilized uniformly during the plant growth phase. RNA was extracted from leaves of the two groups on the 3rd, 5th and 9th days and assessed by qRT-PCR to detect *HvBAHD* gene expression. Changes in the growth of plants were documented. Barley seedling was treated with 10^-4^ M gibberellin for spraying, and the control group was treated with water. All descibed methods including the collection of the barley seeds were performed according to the relevant guidelines and regulations of China.

### Quantitative real-time PCR (qRT-PCR) analysis

Total RNA was isolated from various barley tissues using total RNA Kit trnzol reagent (Tiangen, Beijing, China) following the manufacturer’s protocol. The quality and quantity of all the RNA samples were verified with a Nanodrop 1000 UV spectrophotometer (ThermoFisher Scientific, UT) and checked by 1% agarose gel electrophoresis. For qRT-PCR, first-strand cDNA was reverse transcribed from total RNA with the PrimeScript™ RT reagent Kit with gDNA Eraser (Perfect Real Time; TaKaRa). Real-time RT-PCR was performed with Hieff® qPCR SYBR Green Master Mix (Yeasen, Shanghai, China), using the real-time PCR system (Roche Light Cycler 96). The reaction procedure was a two-step method. The results of relative gene expression were calculated by 2^ΔΔCt^ and the significance was analyzed by t-test^66^. The *HvACTIN* (HORVU1Hr1G074350.1) was used as the internal reference gene^67^. Primers used to quantify the expression of *HvBAHDs* in this study are listed (Supplementary Table S5).

### Subcellular localization of the *HvBAHD* genes in barley

For transient expression in tobacco (*Nicotiana benthamiana*) leaves, full-length *HvBAHD* CDS were PCR amplified using *HvBAHDs*-CDS-F and *HvBAHDs*-CDS-R (sequences in Supplementary Table S5), and introduced into the pCAMBIA1301-GFP vector digested with Bgl II and Spe I to generate 1301-35Spro:*HvBAHD*CDS-GFP. Constructed vectors were transformed into Agrobacterium strain GV3101 and infiltrated into leaves of 4-week-old tobacco plants. After 72 h dark culture, the leaf epidermis of tobacco with 1301-35Spro:*HvBAHD*CDS-GFP was mounted in water and observed under a confocal microscope (Leica TCS SP5), using empty 1301-GFP as a control.

### Cis-regulatory elements prediction of promoter

The 2000 bp upstream sequence of each barley BAHD-related gene was extracted from the barley genome GFF3 file for cis-regulatory element analysis. The data were screened and uploaded to the PlantCARE^63,68^ website (http://bioinformatics.psb.ugent.be/webtools/plantcare/html/) for cis-regulatory element prediction and subsequently visualized in the R studio software.

### SNP (single nucleotide polymorphism) data of *HvBAHD* genes

In the IPK-SNP Browser (https://bridge.ipk-gatersleben.de/#snpbrowser)^69^, the subgroup of barley "core50" was selected as the research object, and the SNP numbers appearing in the *HvBAHD* gene family were counted.

## Supplementary Information

Supplementary Information.
