# Supplementary material for: Systematic identification and expression profiles of the BAHD superfamily acyltransferases in barley (Hordeum vulgare)
Source: Sci Rep. 2022 Mar 24;12:5063. doi: 10.1038/s41598-022-08983-7 (PMC8948222; doi:10.1038/s41598-022-08983-7)
Supplement: Supplementary file 1 — Supplementary Information. [file 41598_2022_8983_MOESM1_ESM.zip › Supplementary Figure2022.03.04_ESM.docx]

**Systematic identification and expression profiles of the BAHD superfamily acyltransferases in barley (*Hordeum vulgare*)**

**Zhen Yuan^1^, Hongliang Yang^1^, Leiwen Pan^1^, Wenhui Zhao^1^, Lunping Liang^1^, Anicet Gatera^1^, Matthew R. Tucker^2^& Dawei Xu^1*^**

^1^ School of Agronomy, Anhui Agricultural University, Hefei 230036, China.

^2^Waite Research Institute, School of Agriculture, Food and Wine, University of Adelaide, Adelaide, SA 5064, Australia.

***** Correspondence: [xudawei@sjtu.edu.cn](mailto:xudawei@sjtu.edu.cn)

**Supplementary Table S1** Physicochemical properties of *HvBAHD* genes and proteins

**Supplementary Table S2** Motif sequences identified by MEME tools in barley *BAHD* gene family

**Supplementary Table S3** Function of *HvBAHD* cis-acting elements

**Supplementary Table S4** Analysis of *BAHD* duplicated genes from barley

**Supplementary Table S5** List of vectors sequence primers used in this study


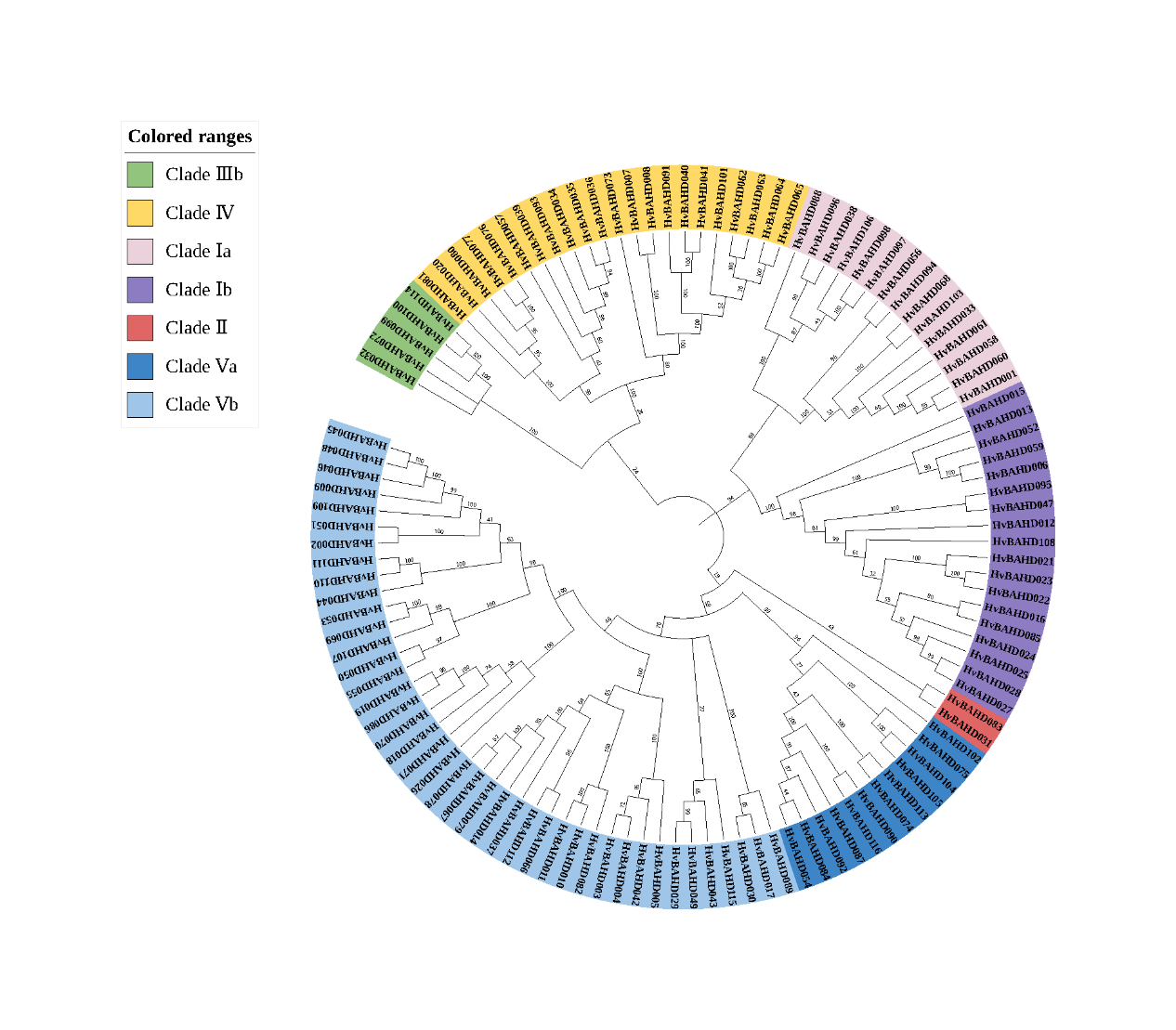


**Supplementary Figure S1.** The phylogenetic relationships among the HvBAHD proteins. This phylogenetic tree was generated by MEGA Ⅹ (https://www.megasoftware.net/) and iTOL online site (https://itol.embl.de/).

**
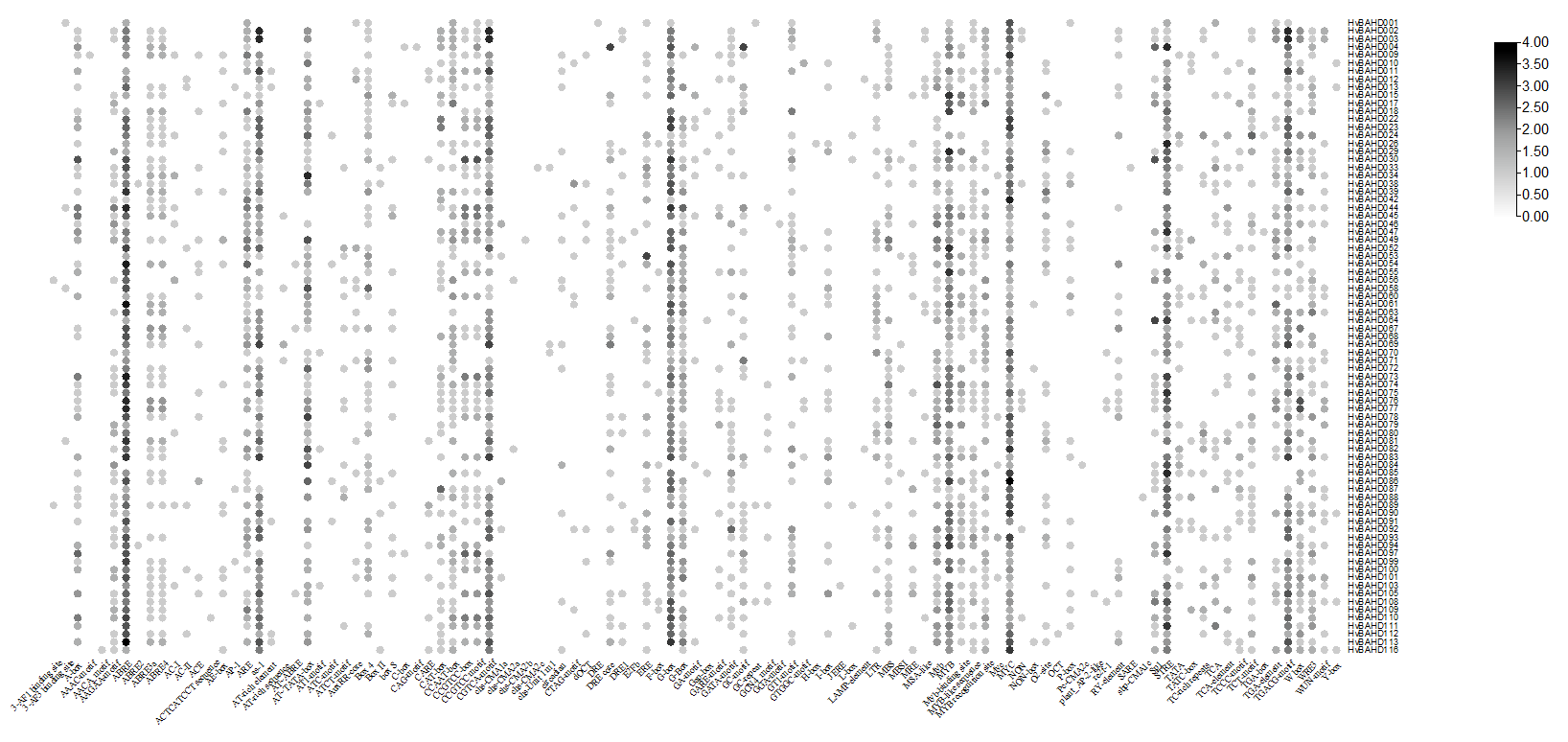
**

**Supplementary Figure S2.** Putative cis-regulatory elements in the 2000bp upstream regions of 79 *HvBAHD* genes in *Hordeum Vulgare*. The CAAT-box and TATA-box components were removed. If the gene presented a dark black dotted band, it indicated that the number and proportion of this element in the barley BAHD family were very high, possibly indicating an important element of the gene family. This picture was constructed by PlantCARE online databases (<http://bioinformatics.psb.ugent.be/webtools/plantcare/html/>) and TBtools v1.082 (https://doi.org/10.1016/j.molp.2020.06.009).


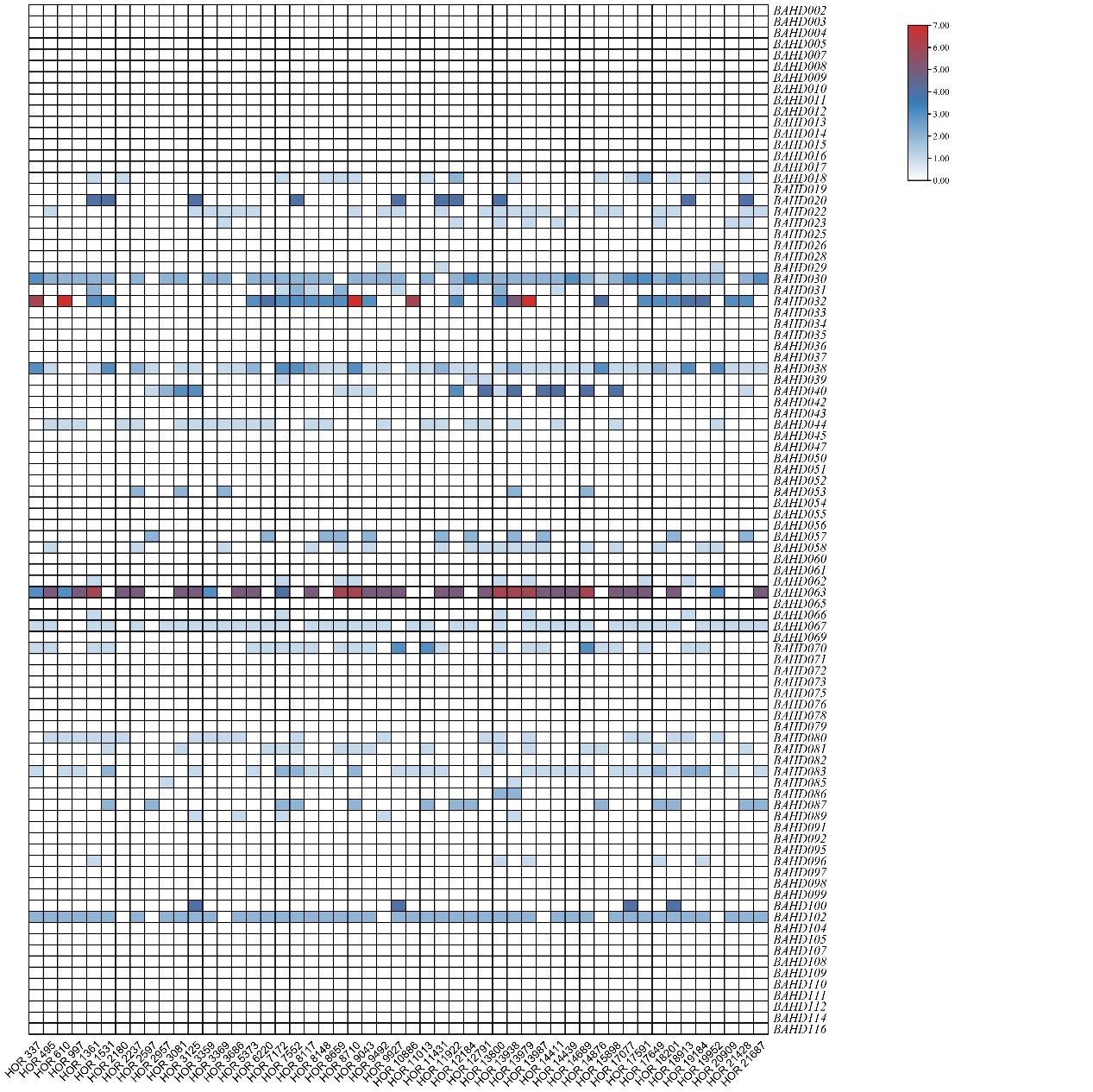


**Supplementary Figure S3.** The numbers of *HvBAHDs* SNP in Different barley Subgroups. The abscissa represents different barley subpopulations. This heatmap was generated by TBtools v1.082 (https://doi.org/10.1016/j.molp.2020.06.009).
