# Supplementary figures and images for: Systematic identification and expression profiles of the BAHD superfamily acyltransferases in barley (Hordeum vulgare)
Source: Sci Rep. 2022 Mar 24;12:5063. doi: 10.1038/s41598-022-08983-7 (PMC8948222; doi:10.1038/s41598-022-08983-7)

## Colored ranges

- 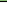 Clade IIIb  
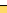 Clade IV  
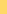 Clade Ia  
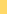 Clade Ib  
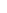 Clade II  
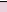 Clade Va  
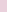 Clade Vb

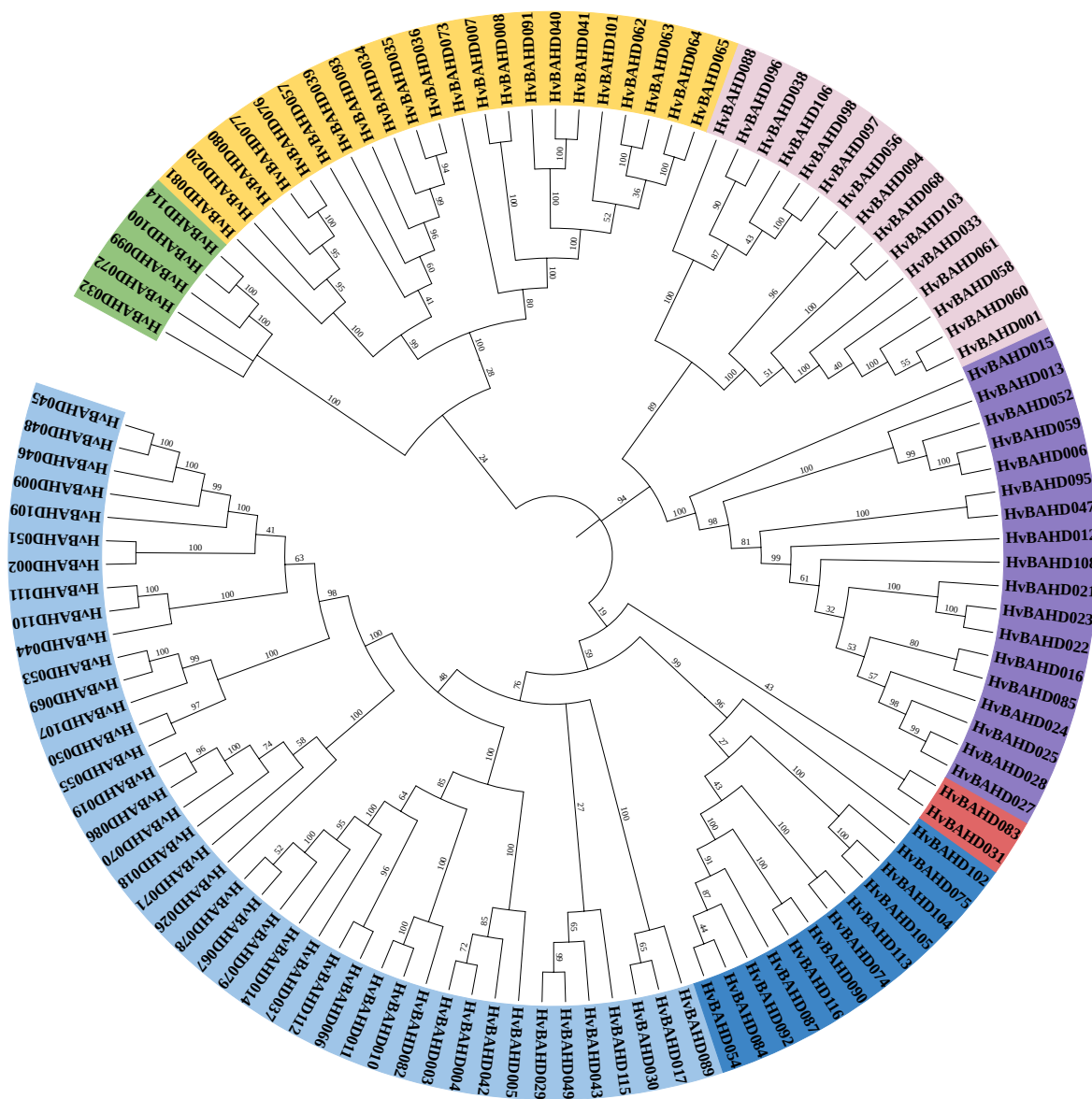

Supplement: Supplementary file 1 — Supplementary Information. [file 41598_2022_8983_MOESM1_ESM.zip › Supplementary Figure S1.pdf]

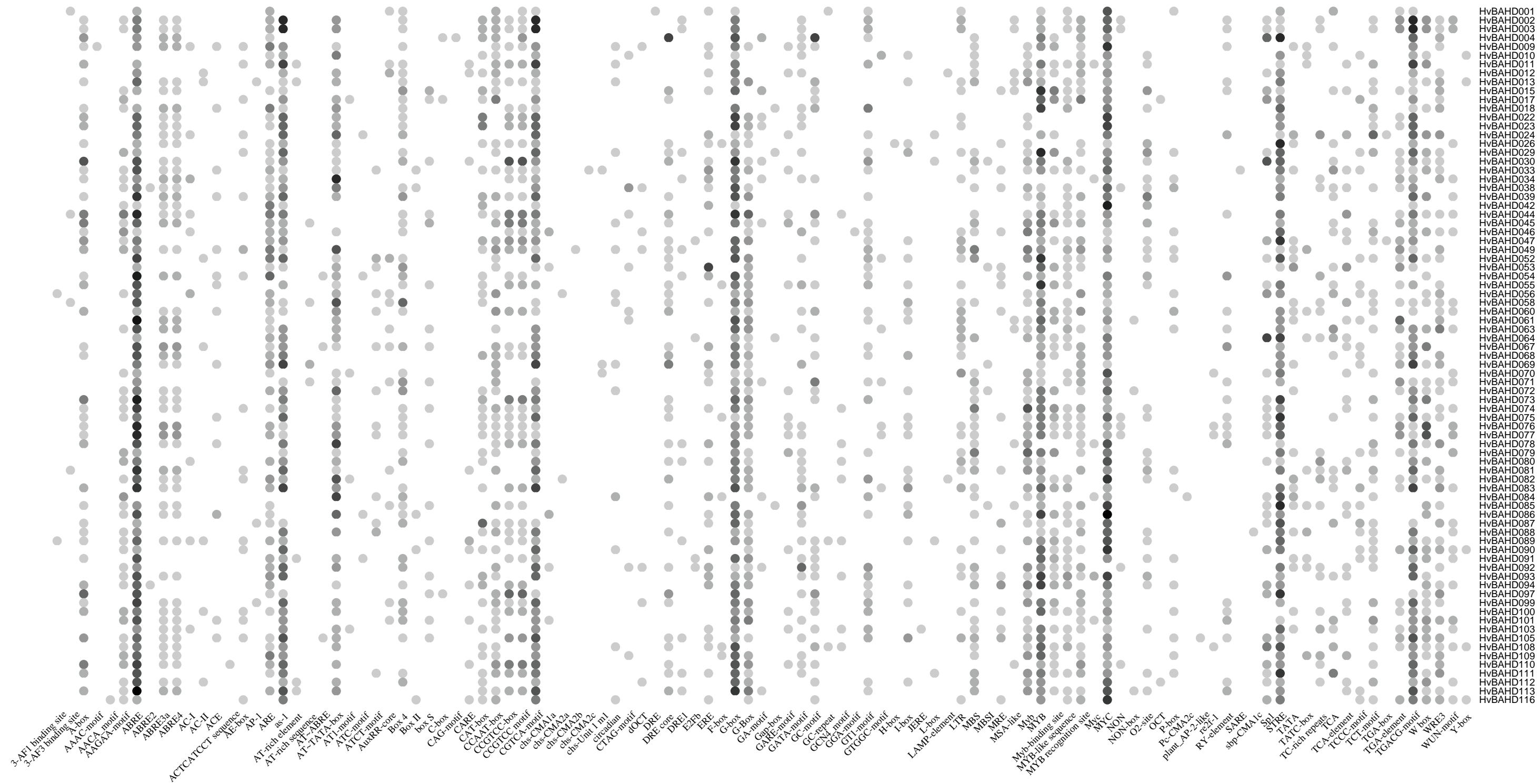

Supplement: Supplementary file 1 — Supplementary Information. [file 41598_2022_8983_MOESM1_ESM.zip › Supplementary Figure S2.pdf]

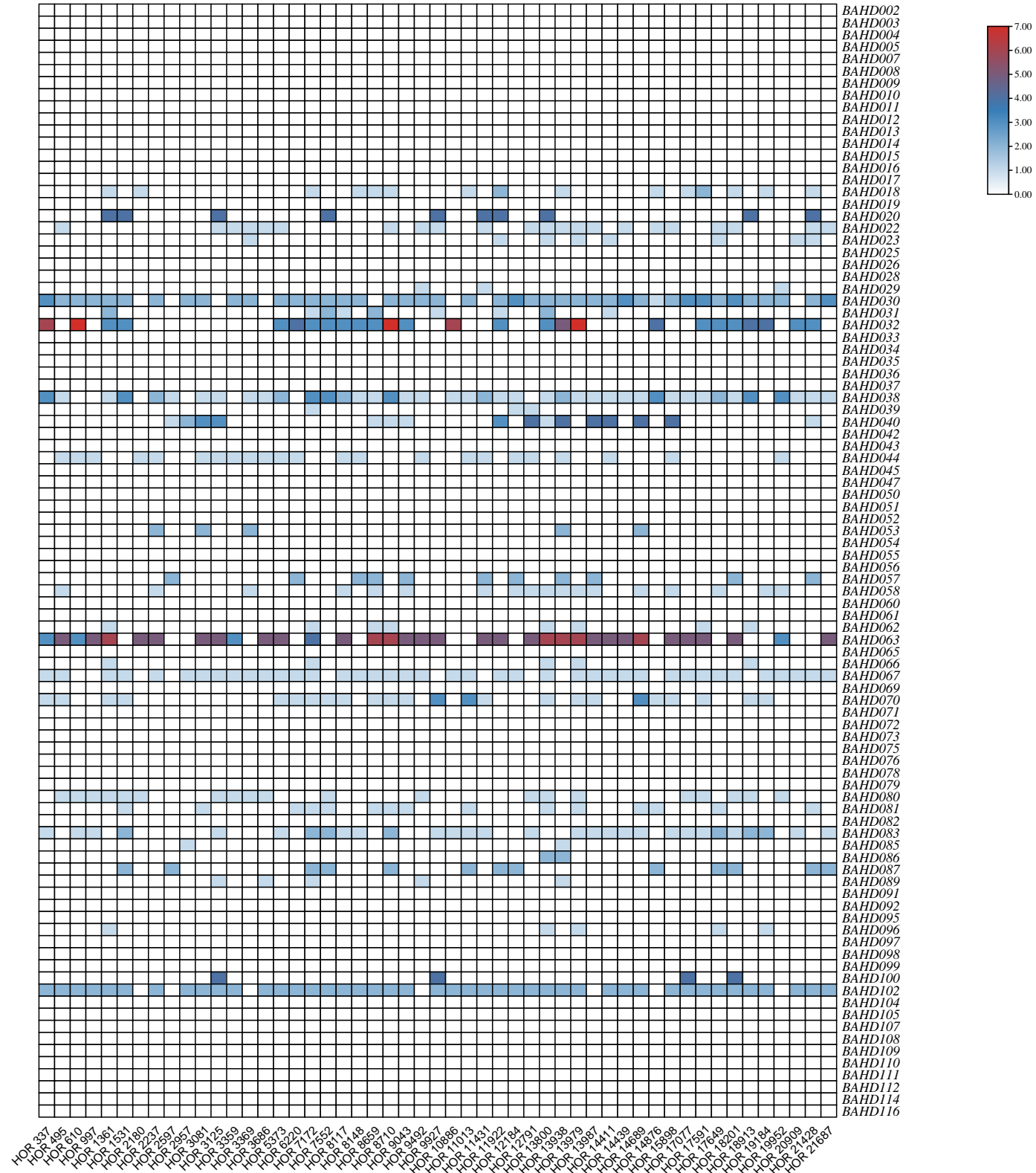

Supplement: Supplementary file 1 — Supplementary Information. [file 41598_2022_8983_MOESM1_ESM.zip › Supplementary Figure S3.pdf]
